# Estimated plasma volume status is a modest predictor of true plasma volume excess in compensated chronic heart failure patients

**DOI:** 10.1038/s41598-021-03769-9

**Published:** 2021-12-20

**Authors:** Christoph Ahlgrim, Philipp Birkner, Florian Seiler, Sebastian Grundmann, Christoph Bode, Torben Pottgiesser

**Affiliations:** 1 Department of Cardiology and Angiology II, Faculty of Medicine, Heart Center Freiburg University, University of Freiburg, Südring 15, 79189 Bad Krozingen, Germany; 2 Department of Cardiology and Angiology I, Faculty of Medicine, Heart Center Freiburg University, University of Freiburg, Freiburg, Germany

**Keywords:** Heart failure, Prognostic markers, Blood flow

## Abstract

Plasma volume and especially plasma volume excess is a relevant predictor for the clinical outcome of heart failure patients. In recent years, estimated plasma volume based on anthropometric characteristics and blood parameters has been used whilst direct measurement of plasma volume has not entered clinical routine. It is unclear whether the estimation of plasma volume can predict a true plasma volume excess. Plasma volume was measured in 47 heart failure patients (CHF, 10 female) using an abbreviated carbon monoxide rebreathing method. Plasma volume and plasma volume status were also estimated based on two prediction formulas (Hakim, Kaplan). The predictive properties of the estimated plasma volume status to detect true plasma volume excess > 10% were analysed based on logistic regression and receiver operator characteristics. The area under the curve (AUC) to detect plasma volume excess based on calculation of plasma volume by the Hakim formula is 0.65 (with a positive predictive value (PPV) of 0.62 at a threshold of − 16.5%) whilst the AUC for the Kaplan formula is 0.72 (PPV = 0.67 at a threshold of − 6.3%). Only the estimated plasma volume status based on prediction of plasma volume by the Kaplan formula formally appears as an acceptable predictor of true plasma volume excess, whereas calculation based on the Hakim formula does not sufficiently predict a true plasma volume excess. The low positive predictive values for both methods suggest that plasma volume status estimation based on these formulas is not suitable for clinical decision making.

## Introduction

The plasma volume (PV) status is a relevant factor when aiming to understand cardiovascular pathophysiology in heart failure patients who often feature a PV overload^1–3^. Guiding heart failure therapy based on direct assessment of volume status can reduce mortality and hospital readmissions due to decompensation in heart failure patients^2^.

Several methods are available to determine blood and plasma volume status in heart failure patients using indicator-dilution techniques based on iodine-labelled Albumin^4^ or carbon monoxide^5^. In compensated heart failure patients, direct measurement of the circulating blood volume allows the detection of a prognostically relevant blood volume expansion, which might otherwise not be recognised^6^. However, the methods measuring blood and plasma volume can be considered as elaborate and have thus not entered the clinical routine.

As an alternative to the direct measurement of plasma volume, several formulas, such as the Kaplan formula^7^ or the so-called Hakim formula^8^ have been proposed to calculate PV from anthropometric characteristics and the haematocrit, a laboratory parameter that is easily available. The resulting “estimated plasma volume” (ePV) has been widely applied when studying heart failure patients and general populations: The plasma volume status (PVS) based on ePV was shown to predict mortality in heart failure patients^9^, early cardiovascular events in heart failure after myocardial infarction^10,11^ and readmission to the hospital after recompensating of decompensated heart failure patients^12,13^. Even procedural outcome after transcatheter aortic valve implantation appears to be affected by the ePV excess on admission^14^. Recently, PVS was shown to be associated with cardiovascular morbidity and mortality in the general population^15^.

Whilst the PVS appears to be related to some echocardiographic and haemodynamic parameters^16^, analyses comparing ePV with the measured “true” PV in heart failure patients has only shown limited association between these parameters^4,9^. It was concluded that the prognostic ability of PVS concerning clinical outcomes of heart failure patients might be independent from directly reflecting the true PV status^4^.

Therefore, from our understanding, the key question is whether a true volume excess can be predicted by PVS on the individual level even when the estimated parameter does not precisely reflect the true volume status, e.g. because of systematic misalignment. Therefore, the aim of this study is to investigate the predictive properties of the calculated PVS based on the Hakim and the Kaplan formula in a group of compensated chronic heart failure patients.

## Methods

### Study design and subjects

The study population has been previously described in more detail^17^. In a cross-sectional design PV was determined in patients with known compensated systolic chronic heart failure and a left ventricular ejection fraction < 50%. 47 patients (10 women) were studied who were recruited from either the outpatient heart failure unit or the cardiology ward (if hospitalised for reasons other than cardiac decompensation). All subjects provided signed informed consent. Exclusion criteria were an acute decompensated state of heart failure (New York Heart Association (NYHA) Class IV), continuous oxygen therapy, haemodynamic instability, acute coronary syndrome, anaemia with a haemoglobin concentration ([Hb]) ≤ 8 g/dl, active bleeding, active malignancy, limited life expectancy < 1 year, noncardiac chronic renal disease, uncontrolled lung disease, chronic inflammatory disease and acute infection. All relevant associated patient data such as medication, functional status, echo and laboratory data were collected in the direct context of the clinic visit.

The study was designed in line with the latest revised form of the Declaration of Helsinki and was approved by the Ethics Committee of the Albert-Ludwigs-University Freiburg (31/14). The study is registered in the German registry for clinical studies (DRKS-ID: DRKS00006078, registered 09/05/2014).

### Determination of plasma volume

The abbreviated CO-rebreathing method (aCORM), an indicator-dilution technique based on rebreathing of carbon monoxide (CO) as a tracer, was applied in all subjects to measure total haemoglobin mass (Hbmass)^18^. Intravascular volumes (red cell volume (RCV), PV and total blood volume (BV)) can then be quantified from aCORM using the following formulas^19^:

$$ {\text{RCV }} = {\text{ Hbmass }}/{\text{ MCHC }} \times {1}00, $$

$$ {\text{BV}} = {\text{RCV}} \times {1}00/{\text{Hct}}^{{1}} , $$

$$ {\text{PV }} = {\text{ BV }}{-}{\text{ RCV}}. $$

MCHC = mean corpuscular haemoglobin concentration, venous [Hb] and Hct were used for determination of MCHC. For RCV calculation, Hct was corrected to whole-body Hct by the factor 0.91^20^ (Hct^1^).

The calculation of true PV excess has been described previously in detail^17^. In brief, we based our analyses on the recommendation of the expert panel of the International Council for Standardization in Haematology (ICSH). The expected normal blood volumes were calculated according to the gender-specific ICSH formulae^21^. In a second step, ICSH estimates were compared with results from aCORM in healthy individuals, thereby normalising the results^17^ leading to an expected normal PV value which takes the application of aCORM into account. A normal volume of PV was then arbitrarily defined as ± 10% of the expected normal value. Consequently, PV excess is a PV > 10% of the expected normal value.

### Estimation of plasma volume

The so-called Hakim^8^ formula and the Kaplan^7^ formulae were used to calculate estimated plasma volume (ePV_method)

$$ {\text{ePV}}\_{\text{Hakim }}\left( {\text{in ml}} \right) \, = \, \left( {{1} - {\text{haematocrit}}} \right) \, \times \, [{\text{a }} + \, \left( {{\text{b }} \times {\text{ body weight }}\left( {\text{in kg}} \right)} \right], $$

a = 1530 in males and 864 in females, and b = 41 in males and 47.9 in females.

$$ {\text{ePV}}\_{\text{Kaplan }}\left( {\text{in ml}} \right) \, = \, \left( {0.0{65 } \times {\text{ body weight }}\left( {\text{in kg}} \right)} \right) \, \times \, \left( {{1 } - {\text{ haematocrit}}} \right) \, \times { 1}000. $$

The ideal PV (iPV) is the normal expected PV calculated based on body weight and sex: iPV (in ml) = c × body weight (in kg).

c = 39 in males and 40 in females^22^. This formula is used as it has been applied in many studies evaluating PVS in order to use the same method despite other, more precise formulas exist^21^.

PVS illustrates whether the ePV_method deviates from iPV and is calculated as: PVS_method = [(ePV_method − iPV)/iPV] × 100%. Values > 100% indicate a higher-than-expected PVS_method.

### Statistical analysis

Data for this study was managed using SAS JMP 9.0, statistical analyses were performed using R^23^. The predicted probabilities of PVS_method to identify subjects with true PV excess were calculated using logistic regression (GLM function, binomial fit). The receiver operator characteristics (ROC) for the respective PVS_method were calculated using the pROC package^24^. The optimal cut-off values of the ROC curve were found using the Youden index^25^. An alpha value of 0.05 was chosen for statistical significance.

## Results

**Figure 1:**
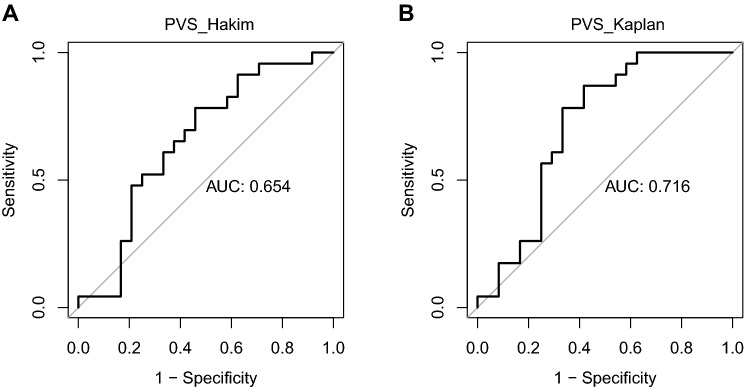
Area-under the curve for the prediction of true plasma volume excess by plasma volume status as calculated by the so-called Hakim formula (**A**) and the Kaplan formula (**B**).

The subject characteristics are described in Table 1. As described previously^17^, true PV excess occurred in 23 subjects. The ROC analysis is depicted in Fig. 1. With an AUC of 0.65, PVS when calculated based on the so-called Hakim formula is a “poor”^26^ predictor of true PV excess assessed by the aCORM (best cut-off: − 16.5%; with a corresponding sensitivity of 0.78, a specificity of 0.54, a positive predictive value of 0.62, and a negative predictive value of 0.72). With an AUC of 0.72, PVS based on the Kaplan formula formally appears as an acceptable^26^ predictor of true PV excess (best cut-off value: − 6.3%; with a corresponding sensitivity of 0.87, a specificity of 0.58, a positive predictive value of 0.67, and a negative predictive value of 0.82).

**Table 1:** Baseline characteristics (n = 47) as in part previously published^17^.

Age (years)	57.8 ± 8.9
Weight (kg)	83.3 ± 17.6
Blood pressure (mmHg)	122 ± 18/76 ± 11
proBNP (pg/ml)	2628 ± 5219 (n = 37)
Left atrial diameter	46 ± 9
Left ventricular end-diastolic diameter (mm)	66 ± 12
Relative wall thickness	0.33 ± 0.09 (n = 46)
Left-ventricular mass index (g/m^2^)	157 ± 49 (n = 46)
Ejection fraction (%), mod. Simpson rule	29.0 ± 9.4 (n = 41)
**Cause of CHF**	
Ischaemic CM, n (%)	21 (45)
Dilated CM, n (%)	19 (40)
Myocarditis, n (%)	3 (7)
Valvular CM, n (%)	2 (4)
Hypertensive CM, n (%)	1 (2)
Plasma volume (ml)	4089 ± 960
Plasma volume excess, n (%)	23 (49)
ePV_Kaplan (ml)	3096 ± 594
ePV_Hakim (ml)	2805 ± 404
PVS_Kaplan (%)	− 4.5 ± 6.4
PVS_Hakim (%)	− 12.5 ± 9.0
**Medication**	
Beta blocker, n (%)	44 (94)
Ivabradin, n (%)	4 (9)
ACE-inhibitor, n (%)	31 (66)
AT1-antagonist, n (%)	12 (26)
Any diuretic therapy, n (%)	33 (70)
Loop diuretic, n (%)	31 (70)
Thiazide, n (%)	7 (15)
Other diuretic, n (%)	3 (7)
Mineralcorticoid receptor antagonist	36 (77)
Sum of diuretics	1.6 ± 0.9

## Discussion

Recognition of blood volume excess is challenging as it might occur independent from physical signs of congestion but drives cardiovascular outcomes^6^. Our own observations in clinically compensated heart failure patients confirm the presence of considerable heterogeneity in the blood volume status^17^, which, on the other hand, appears to be relatively preserved over time on the individual level in stable patients, with PV changing 1.3% on average when measured repeatedly over the span of 6 months using the same method as it was applied here^27^. Furthermore, the typical measurement error for PV quantified by aCORM is between 3.8 and 4.7% when measured twice within a week in healthy subjects^28^. The heterogeneity of volume status imposes a diagnostical challenge when aiming to detect plasma volume excess but may also provide new options for management of heart failure patients: Measuring or estimating the circulating blood volume at different time points and the comparison of these changes with clinical indices and biomarkers in patients with chronic heart failure could be of clinical utility in terms of long-term management of this progressive chronic disease and evaluation of treatment effects.

Authors have previously studied the association between estimated PVS and echocardiographic and haemodynamic parameters related to congestive heart failure but reported heterogeneous results: Kobayashi et al. were able to establish a connection between estimated PVS and diastolic filling pressures and the E/E′ ratio in heart failure patients whilst vena cava width and ultrasonographic signs of congestions were unrelated to estimated PVS^16^.

To our knowledge, this is the first report to evaluate the predictive properties of the estimated PVS concerning the prediction of a ‘true’ measured PV excess in a cohort of patients with chronic heart failure. In our study, we could show that PVS calculated from the Kaplan formula based on anthropometric and hematologic data is an acceptable predictor of true PV excess whereas the so-called Hakim formula insufficiently predicts a true PV excess. However, while a ROC of 0.72 is formally considered as an “acceptable” predictive property^26^, this value is not acceptable in a clinical situation where higher odds than 72% are required to distinguish between a normal and a pathological status: our analyses concerning the positive predictive value of PVS_Kaplan show that one in three patients with a PVS >  − 6.3% (the best-cut off value) does not feature a true PV excess of > 10%.

In addition to the work presented by Fudim and Miller^4^, who could show that the agreement between measured and calculated values for PV is moderate at best, our data therefore illustrate that even when the calculated PVS is considered as a predictor for an individual’s PV excess and is used solely for this purpose, there is only limited clinical value of the parameter in this context.

This is remarkable from a (patho)physiological point of view given the predictive properties of the (estimated) PVS for cardiovascular outcomes^9,10,12^, which are unchallenged by our study. Apparently, PVS predicts cardiovascular outcomes, such as rehospitalisation, morbidity and mortality in patients stratified by this parameter whilst featuring only a modest connection to the true plasma volume of the individual patient. Therefore, patients that are identified as having an excess PVS by calculated parameters are not necessarily or exclusively those with a true PV excess.

Independent from an association with true PV, both, estimated PV and PVS could be considered as a compound risk predictor. When analysing the composition of estimated PV and PVS, it appears that the parameters depend on haematocrit, body weight and sex. At least haematocrit^29^ and sex^30^ have been shown to affect the cardiovascular prognosis. Moreover, when dry body weight is used to calculate the estimated PV by the so-called Hakim formula and, within the same mathematical approach, the ideal PV is calculated using normal body weight^9^, an indicator for body fat content is created, which might also predict cardiovascular prognosis^31,32^ and is associated with diastolic filling pressure^33^.

In this context, it is worth looking at the methodological history of the formula that has frequently been referred to as the Hakim-formula as it is mentioned in a book chapter written by Ismail et al.^8^. The formula, however, is based on much older work of Brown et al. and Wennesland et al. describing regression formulae for the total blood volume based on weight in women^34^ and men^35^ derived from blood volume measurements using 51Cr labelled erythrocytes. The term ‘1-haematocrit’ was added later to calculate the PV fraction from the total blood volume. This is, however, an oversimplification without taking the so called body-venous-haematocrit factor^20^ into account and thus, systematically underestimates PV. In this context, it is interesting that a minority of authors have applied the so-called Hakim formula using lean body weight^4,12^, as specifically recommended by Hakim, whereas others have applied the formula using the uncorrected raw body mass^9,13–15,36^ as it was intended in the original blood volume formula by Brown et al. and Wennesland et al.^34,35^. Efforts to validate the so-called Hakim Formula in heart failure patients unsurprisingly yielded only moderate correlation coefficients between ePV and measured PV with a systematic deviation of the formula in subjects with higher PV^4,9^. The work by Sprenger et al., taking deviations in haematocrit from the normal value into account, aims to overcome some of the drawbacks discussed above and also addresses the body-venous-haematocrit ratio^37^ and might be a more suitable approach to estimate PV, although this approach has not been validated in heart failure patients.

Concerning the ideal PVS, other, more elaborate algorithms predicting the expected normal PV based on body surface area and sex have been proposed before by the International Council for Standardization in Haematology to avoid body fat mass as a confounding variable^21^. This approach was also chosen by our group when calculating PV excess from PV measurements^17^, which is used as the reference parameter here.

In summary, these methodological aspects might further contribute to the predictive properties of the “calculated PVS” concerning cardiovascular outcomes and the relation to the diastolic filling pressures of the heart independent from predicting a true PV excess.

As discussed above, the haematocrit value affects prognosis of heart failure patients and is closely related to the PVS. Although this value is ubiquitously available, it could be considered more by the clinician and put in the overall context over time. Thus, it may be prudent to regard a decreasing haematocrit value of a patient as an indicator of PV excess. It still needs to be defined, if the possible consequence of increasing the dose of diuretics in such a case is sufficient and beneficial to the patient, especially in the light of the recently available inhibitors of sodium dependent glucose co-transporter 2.

## Conclusion

Therefore, from our perspective and in agreement with Fudim and Miller^4^, we believe PVS as calculated from the formulas evaluated here should not be taken into account when aiming at decision making on the individual patient level concerning PV management. More work is necessary to clarify the origin of the predictive properties of calculated estimates of volume status for clinical outcomes of heart failure patients.

## References

[1] Adlbrecht C, Kommata S, Hülsmann M, Szekeres T, Bieglmayer C, Strunk G, Karanikas G, Berger R, Mörtl D, Kletter K, Maurer G, Lang IM, Pacher R (2008). Chronic heart failure leads to an expanded plasma volume and pseudoanaemia, but does not lead to a reduction in the body’s red cell volume. Eur. Heart J..

[2] Strobeck JE, Feldschuh J, Miller WL (2018). Heart failure outcomes with volume-guided management. JACC Heart Fail..

[3] Miller WL (2016). Fluid volume overload and congestion in heart failure: Time to reconsider pathophysiology and how volume is assessed. Circ. Heart Fail..

[4] Fudim M, Miller WL (2018). Calculated estimates of plasma volume in patients with chronic heart failure—Comparison to measured volumes. J. Card. Fail..

[5] Ahlgrim C, Birkner P, Seiler F, Grundmann S, Baumstark MW, Bode C, Pottgiesser T (2018). Applying the optimized CO rebreathing method for measuring blood volumes and hemoglobin mass in heart failure patients. Front. Physiol..

[6] Androne AS, Hryniewicz K, Hudaihed A, Mancini D, Lamanca J, Katz SD (2004). Relation of unrecognized hypervolemia in chronic heart failure to clinical status, hemodynamics, and patient outcomes. Am. J. Cardiol..

[7] Kaplan AA (1990). A simple and accurate method for prescribing plasma exchange. ASAIO Trans..

[8] Ismail N, Neyra R, Hakim RM, Daugirdas JT, Blake PG, Ing TS (2001). Plasmapheresis. Handbook of Dialysis.

[9] Ling HZ, Flint J, Damgaard M, Bonfils PK, Cheng AS, Aggarwal S, Velmurugan S, Mendonca M, Rashid M, Kang S, Papalia F, Weissert S, Coats CJ, Thomas M, Kuskowski M, Cohn JN, Woldman S, Anand IS, Okonko DO (2015). Calculated plasma volume status and prognosis in chronic heart failure. Eur. J. Heart Fail..

[10] Duarte K, Monnez J-M, Albuisson E, Pitt B, Zannad F, Rossignol P (2015). Prognostic value of estimated plasma volume in heart failure. JACC Heart Fail..

[11] Kawai T, Nakatani D, Yamada T, Sakata Y, Hikoso S, Mizuno H, Suna S, Kitamura T, Okada K, Dohi T, Kojima T, Oeun B, Sunaga A, Kida H, Sato H, Hori M, Komuro I, Tamaki S, Morita T, Fukunami M, Sakata Y (2021). Clinical impact of estimated plasma volume status and its additive effect with the GRACE risk score on in-hospital and long-term mortality for acute myocardial infarction. IJC Heart Vasc..

[12] Tamaki S, Yamada T, Morita T, Furukawa Y, Iwasaki Y, Kawasaki M, Kikuchi A, Kawai T, Seo M, Abe M, Nakamura J, Yamamoto K, Kayama K, Kawahira M, Tanabe K, Ueda K, Kimura T, Sakamoto D, Fukunami M (2019). Prognostic value of calculated plasma volume status in patients admitted for acute decompensated heart failure—A prospective comparative study with other indices of plasma volume. Circ. Rep..

[13] Grodin JL, Philips S, Mullens W, Nijst P, Martens P, Fang JC, Drazner MH, Tang WHW, Pandey A (2019). Prognostic implications of plasma volume status estimates in heart failure with preserved ejection fraction: Insights from TOPCAT. Eur. J. Heart Fail..

[14] Maznyczka AM, Barakat M, Aldalati O, Eskandari M, Wollaston A, Tzalamouras V, Dworakowski R, Deshpande R, Monaghan M, Byrne J, Wendler O, MacCarthy P, Okonko D (2020). Calculated plasma volume status predicts outcomes after transcatheter aortic valve implantation. Open Heart.

[15] Otaki Y, Watanabe T, Konta T, Watanabe M, Asahi K, Yamagata K, Fujimoto S, Tsuruya K, Narita I, Kasahara M, Shibagaki Y, Iseki K, Moriyama T, Kondo M, Watanabe T (2020). Impact of calculated plasma volume status on all-cause and cardiovascular mortality: 4-year nationwide community-based prospective cohort study. PLoS ONE.

[16] Kobayashi M, Huttin O, Donal E, Duarte K, Hubert A, Le Breton H, Galli E, Fournet M, Mabo P, Schnell F, Leclercq C, Rossignol P, Girerd N (2020). Association of estimated plasma volume status with hemodynamic and echocardiographic parameters. Clin. Res. Cardiol..

[17] Ahlgrim C, Birkner P, Seiler F, Wrobel N, Grundmann S, Bode C, Pottgiesser T (2020). Increased red cell volume is a relevant contributing factor to an expanded blood volume in compensated systolic chronic heart failure. J. Card. Fail..

[18] Schmidt W, Prommer N (2005). The optimised CO-rebreathing method: A new tool to determine total haemoglobin mass routinely. Eur. J. Appl. Physiol..

[19] Heinicke K, Wolfarth B, Winchenbach P, Biermann B, Schmid A, Huber G, Friedmann B, Schmidt W (2001). Blood volume and hemoglobin mass in elite athletes of different disciplines. Int. J. Sports Med..

[20] Chaplin H, Mollison PL, Vetter H (1953). The body/venous hematocrit ratio: Its constancy over a wide hematocrit range. J. Clin. Investig..

[21] Pearson TC, Guthrie DL, Simpson J, Chinn S, Barosi G, Ferrant A, Lewis SM, Najean Y (1995). Interpretation of measured red cell mass and plasma volume in adults: Expert panel on radionuclides of the International Council for Standardization in Haematology. Br. J. Haematol..

[22] Longo DL, Jameson JL, Kaspe D (2011). Harrison’s Principles of Internal Medicine.

[23] Team RC. *R: A Language and Environment for Statistical Computing* (2013).

[24] Robin X, Turck N, Hainard A, Tiberti N, Lisacek F, Sanchez J-C, Müller M (2011). pROC: An open-source package for R and S+ to analyze and compare ROC curves. BMC Bioinform..

[25] Youden WJ (1950). Index for rating diagnostic tests. Cancer.

[26] Hosmer DW, Lemeshow S, Sturdivant RX (2013). Applied Logistic Regression.

[27] Ahlgrim C, Seiler F, Birkner P, Staudacher DL, Grundmann S, Bode C, Pottgiesser T (2021). Time course of red cell volume and plasma volume over six months in compensated chronic heart failure. ESC Heart Fail..

[28] Bourdon P, Lobigs L, Nikolovski Z, El-Gingo M, Varamenti E, Schumacher YO (2015). Reliability and accuracy of plasma volume measures using two different analysers: 789 Board# 185 May 27, 2: 00 PM-3: 30 PM. Med. Sci. Sports Exerc..

[29] Guglin M, Darbinyan N (2012). Relationship of hemoglobin and hematocrit to systolic function in advanced heart failure. Cardiology.

[30] Magnussen C, Niiranen TJ, Ojeda FM, Gianfagna F, Blankenberg S, Vartiainen E, Sans S, Pasterkamp G, Hughes M, Costanzo S, Donati MB, Jousilahti P, Linneberg A, Palosaari T, de Gaetano G, Bobak M, den Ruijter HM, Jørgensen T, Söderberg S, Kuulasmaa K, Zeller T, Iacoviello L, Salomaa V, Schnabel RB (2019). Sex-specific epidemiology of heart failure risk and mortality in Europe: Results from the BiomarCaRE Consortium. JACC Heart Fail..

[31] Oreopoulos A, Ezekowitz JA, McAlister FA, Kalantar-Zadeh K, Fonarow GC, Norris CM, Johnson JA, Padwal RS (2010). Association between direct measures of body composition and prognostic factors in chronic heart failure. Mayo Clin. Proc..

[32] Oreopoulos A, Padwal R, Kalantar-Zadeh K, Fonarow GC, Norris CM, McAlister FA (2008). Body mass index and mortality in heart failure: A meta-analysis. Am. Heart J..

[33] Russo C, Jin Z, Homma S, Rundek T, Elkind MSV, Sacco RL, Di Tullio MR (2011). Effect of obesity and overweight on left ventricular diastolic function: A community-based study in an elderly cohort. J. Am. Coll. Cardiol..

[34] Brown E, Hopper J, Hodges JL, Bradley B, Wennesland R, Yamauchi H (1962). Red cell, plasma, and blood volume in the healthy women measured by radiochromium cell-labeling and hematocrit. J. Clin. Investig..

[35] Hopper J, Hodges JL, Guttentag OE, Scott KG, Tucker IN, Bradley B (1959). Red cell, plasma and blood volume in healthy men measured by radiochromium (Cr51) cell tagging and hematocrit: Influence of age, somatotype and habits of physical activity on the variance after regression of volumes to height and weight combined. J. Clin. Investig..

[36] Kobayashi M, Girerd N, Duarte K, Preud’homme G, Pitt B, Rossignol P (2020). Prognostic impact of plasma volume estimated from hemoglobin and hematocrit in heart failure with preserved ejection fraction. Clin. Res. Cardiol..

[37] Sprenger KBG, Huber K, Kratz W, Henze E (1987). Nomograms for the prediction of patient’s plasma volume in plasma exchange therapy from height, weight, and hematocrit. J. Clin. Apheresis.

